# The immunomodulator decoy receptor 3 improves locomotor functional recovery after spinal cord injury

**DOI:** 10.1186/s12974-016-0623-6

**Published:** 2016-06-17

**Authors:** Chuan-Wen Chiu, Wen-Hung Huang, Shao-Ji Lin, May-Jywan Tsai, Hsu Ma, Shie-Liang Hsieh, Henrich Cheng

**Affiliations:** Department and Institute of Pharmacology, National Yang-Ming University, Taipei, 11221 Taiwan; Neural Regeneration Laboratory, Taipei, 11217 Taiwan; Center for Neural Regeneration, Department of Neurosurgery, Neurological Institute, Taipei Veterans General Hospital, Taipei, 11217 Taiwan; Division of Plastic and Reconstructive Surgery, Department of Surgery, Taipei Veterans General Hospital, Taipei, 11217 Taiwan; Genomics Research Center, Academia Sinica, Taipei, Taiwan; Institute of Clinical Medicine, National Yang-Ming University, Taipei, Taiwan; Department of Medical Research, Taipei Veterans General Hospital, Taipei, Taiwan

**Keywords:** Spinal cord injury, DcR3, Alternatively activated macrophage

## Abstract

**Background:**

Spinal cord injury (SCI) causes loss of neurons and axons and results in motor and sensory function impairments. SCI elicits an inflammatory response and induces the infiltration of immune cells, predominantly macrophages, to the injured site. Decoy receptor 3 (DcR3), also known as tumor necrosis factor receptor superfamily member (TNFRSF)-6B, is a pleiotropic immunomodulator capable of inducing macrophage differentiation into the M2 phenotype and enhancing angiogenesis. Because M2 macrophages are crucial for the recovery of impaired motor functions, we ask whether DcR3 is beneficial for the functional recovery of locomotion in Sprague-Dawley (SD) rats after SCI.

**Methods:**

Contusion injury of the spinal cord was performed using a New York University impactor at the ninth thoracic vertebrae, followed by intrathecal injection of 15 μg recombinant protein comprising DcR3 (DcR3.Fc) in 5 μl of normal saline as the treatment, or 5 μl of normal saline as the control, into the injury epicenter. Functional recovery was evaluated using an open-field test weekly up to 6 weeks after injury. The cavity size and myelin sparing in the rostral-to-caudal region, including the epicenter of the injury, were then examined in SCI rats by histological staining. The expression of anti-inflammatory cytokines and the presence of M2 macrophages were determined by quantitative real-time polymerase chain reaction (qPCR) and immunohistochemistry at 7 day after SCI. Statistical analysis was performed using a two-tailed Student’s *t* test.

**Results:**

Intrathecal administration of DcR3.Fc significantly improved locomotor function and reduced secondary injury with a smaller wound cavity and increased myelin sparing at the lesion site. Compared with the control group, DcR3.Fc-treated rats had increased vascularization at the injury epicenter along with higher levels of interleukin (IL)-4 and IL-10 and lower level of IL-1β on DcR3.Fc-treated rats at day 7 after SCI. Moreover, higher levels of arginase I (Arg I) and CD206 (M2 macrophage markers) and RECA-1 (endothelial marker) were observed in the epicenter on day 7 after SCI by immunofluorescence staining.

**Conclusions:**

These results indicated that DcR3.Fc may promote the M2 macrophage infiltration and enhanced angiogenesis at the lesion site, thus preserving a greater amount of spinal cord tissues and enhancing functional recovery after SCI.

## Background

Spinal cord injury (SCI) often causes incurable neurological dysfunction due to failure of axonal regeneration in adult mammals. SCI causes devastating damage to the affected individuals, and patients’ families suffer from significant financial loss and psychological stress. Therefore, therapeutic agents capable of improving locomotor functional recovery would alleviate the suffering of the affected individuals and reduce the socioeconomic burden. Despite decades of research, an effective therapeutic reagent for severe SCI remains elusive, and the current treatment is limited to the early administration of high-dose steroids and acute surgical intervention to minimize cord edema and the neuronal injuries [1]. The pathophysiological processes of SCI comprise of multiple phases. During traction injury, compression forces and direct mechanical disruption of neural elements are common initial physical traumas to the spinal cord. Microvascular hemorrhage with disruption of the blood-spinal cord barrier is followed by edema, ischemia, and the release of cytotoxic chemicals from inflammatory pathways. The secondary injury cascade that compounds the initial mechanical injury with cell necrosis and apoptosis further exacerbates the initial damage and expands the lesion area [2]. Moreover, secondary neurodegenerative events (including demyelination, Wallerian degeneration, and axonal dieback) occur in the non-permissive tissue environment due to astroglial scar formation, leading to irreversible loss of functions [3, 4]. The inflammatory response in the secondary phase after SCI is a series of complex cellular and molecular interactions, further reducing the opportunity for recovery of penumbral neurons and rendering functional recovery almost hopeless [5, 6]. SCI induces infiltration of immune cells, predominantly macrophages, to the injured sites. In the past two decades, intensive studies have elucidated the critical roles of macrophages in the central nervous system (CNS) pathologies, ranging from acute insults to neurodegenerative diseases [7]. Thus, modulation of macrophage activity to create a more permissive environment for tissue repair would benefit patients suffering from SCI and other neurodegeneration diseases.

Macrophages are heterogeneous cell populations with distinct ability in tissue repair after injury. Post-injury CNS repair occurs via the balance of two major populations of macrophages: the pro-inflammatory type I macrophages (M1) and the type II anti-inflammatory (M2) macrophages [8]. Whether macrophages aggravate secondary injury or promote wound repair depends on the signals present in the microenvironment that skew the cell differentiation into M1 or M2 macrophages [8–11]. M1 macrophages respond to pro-inflammatory cytokines, such as interferon-gamma, and kill pathogens efficiently. However, M1 macrophages produce high levels of nitric oxide, pro-inflammatory cytokines, and matrix metalloproteinases, which frequently cause tissue damage and axonal retraction [12]. In contrast, M2 macrophages respond to anti-inflammatory cytokines, such as IL-4, IL10, and IL-13, which are involved in tissue repair [13]. It has been shown that M2 macrophages can promote axonal growth and overcome inhibitory substrates [14]; thus, we asked whether induction of M2 macrophages after acute neuronal injury will be able to speed up tissue repair and improve functional recovery.

Decoy receptor 3 (DcR3/tumor necrosis factor receptor superfamily member (TNFRSF)-6B), a soluble receptor belonging to the tumor necrosis factor receptor superfamily, has been shown to be a potent pleiotropic immunomodulator that skews macrophage into M2 phenotype and enhances angiogenesis [15]. DcR3 is able to neutralize FasL/TNFSF6 [16], LIGHT/TNFSF14 [17], and TL1A/TNFSF15 [18] to inhibit apoptosis and allogeneic reaction, as well as to promote angiogenesis [19, 20]. Moreover, DcR3 is able to modulate cell functions via activation of the heparan sulfate proteoglycans [21]. In addition to modulating the activation and differentiation of dendritic cells [22, 23], DcR3 is able to skew monocyte differentiation toward M2 macrophages [24], which play crucial roles in angiogenesis and tissue repair [25–27]. Therefore, we asked whether the administration of recombinant protein comprising DcR3 (DcR3.Fc) is able to promote tissue repair and the recovery of locomotor neurons after SCI.

In this study, we investigated the potential therapeutic effects of DcR3.Fc in SCI rats. We found that DcR3.Fc promoted locomotor functional recovery and enhanced tissue repair in SCI rats after contusive SCI. Moreover, increased infiltration of M2 macrophage was noted with enhanced angiogenesis in the epicenter of the lesion site. Furthermore, a higher expression of anti-inflammatory cytokines with downregulation of inflammatory cytokines was observed in the injury epicenter. All these observation suggested that DcR3.Fc has the potential to become a therapeutic agent for SCI in the future.

## Methods

### Production of recombinant human DcR3 protein

DcR3.Fc was produced as previously described [22]. Briefly, Sf21 cells were used to amplify and produce DcR3.Fc protein. The supernatant from recombinant virus-infected Sf21 cells was filtered and purified on protein A-Sepharose beads. The bound DcR3.Fc proteins were eluted with 0.1 M glycine buffer (pH 3.0), followed by dialysis against phosphate-buffered saline (PBS).

### Animal surgery

Adult female Sprague-Dawley (SD) rats (225–250 g) were used in this study. All procedures involving animals were approved by the Animals Committee of Taipei Veterans General Hospital (permit numbers IACUC 2013-096 and IACUC 2014-137) and were in accordance with the Guide for the Care and Use of Laboratory Animals outlined by the National Institutes of Health. SCI was induced using the New York University (NYU) weight-drop device as previously described [28]. Briefly, rats were anesthetized and underwent a T8–10 laminectomy. A 10-g steel rod was allowed to drop 5 cm onto the exposed dura at the T9 vertebral level to produce a contusion injury. An intraspinal injection of DcR3.Fc (15 μg) in 5 μl of normal saline or 5 μl of normal saline was administered after contusion production through a 5-μl Hamilton syringe with a 33-gauge needle. The needle was held for 5 min after injection and then slowly withdrawn from the spinal cord. Manual emptying of the bladder was performed twice daily.

### Behavioral testing

The Basso, Beattie, and Bresnahan (BBB) open-field score was used to evaluate locomotion in terms of hind limb functional improvement of the rats with SCI [29]. The BBB test was scored from 0 (no observable hind limb movement) to 21 (normal hind movement) points. In this study, behavioral analyses were conducted every week after surgery for 6 weeks. The behavioral tests were recorded by a video camera, and both examiners were blinded to the behavioral evaluation group.

### RNA isolation, reverse transcription, and quantitative real-time PCR

At 7 days post-injury, 5 mm of tissue from the lesion site in the spinal cord was preserved by Allprotect Tissue Reagent (QIAGEN®, Darmstadt, Germany) and homogenized with the MagNA Lyser Instrument (Roche®, Penzberg, Germany), as described previously [10]. Total RNA and proteins were extracted following the suggested protocol of the AllPrep® DNA/RNA/Protein Mini Kit (QIAGEN®, Darmstadt, Germany). cDNA synthesis was primed with oligo dT, followed by reverse transcription using a Reverse Transcriptase kit (QIAGEN®). cDNA levels were quantified using primer pairs and FastStart Universal SYBR Green Master (Roche) on a StepOne™ Real-Time PCR System (Applied Biosystems). Glyceraldehyde 3-phosphate dehydrogenase (GAPDH, F-5′ AGGTGGTGGTTG TACGCTGTG 3′, R-5′ TGAACTTGCCGTGGGTAGAG 3′) was used as an internal control for normalization. All PCR reactions were performed in triplicates, and the specificity of the reaction was determined using melting curve analysis at the dissociation stage. All PCR primer sets had been validated and tested for efficiency. Both positive and negative controls were included on the same plate for qPCR. The relative differences in expression between groups were analyzed on the basis of cycle values using the comparative threshold cycle (Ct) method [30], in which Ct is the cycle exhibiting the first detectable increase in SYBR green fluorescence above the threshold. The target gene quantity was normalized to a reference gene using the following formula: 2^−(Ct(target) − Ct(reference))^.

### Quantitative analysis of the cavity area and myelinated white matter

At 6 weeks post-injury, rats were intravascularly perfused with 0.9 % saline and 4 % paraformaldehyde in PBS. The spinal cords were removed from the experimental animal, post-fixed overnight in 4 % PFA, rinsed, cryoprotected in graded sucrose, and embedded into the optimal cutting temperature compound at −20 °C. Samples were transversely sectioned (20 μm thick) and then placed on slides for staining and quantitation as described previously [31]. Briefly, hematoxylin and eosin (H&E) was used to stain nuclei and eosin for cavities and Luxol fast blue (LFB) used to identify myelinated white matter. Images were photographed from the rostral end to the caudal end throughout the injury site at ×2.5 magnification with a microscope camera (20 sections per animal, 200 μm between sections). For quantification, the images of H&E staining were converted to gray scale (0–255 levels such that 0 = black pixel, 255 = white pixel), and the total cord area (0–255) and the optimized threshold values of brightness (≥225) as cavity area in each section were determined by using the ImageJ 1.44d software (Wayne Rasband, National Institutes of Health, Bethesda, MD, USA). The cavity area was calculated as a percentage of the total cord area on each section, and the section with the highest cavity area was assumed to be the central position of the injury. LFB-positive area was measured as the myelinated area. After the myelinated area in each section was determined, the Cavalieri method [32] was used to calculate the total volume by summing their individual subvolumes. Individual subvolumes were calculated by multiplying the myelinated area (*A*) × *D*, where *D* represents the distance between sections (200 μm).

### Western blot analysis

Protein lysates from the spinal cords were centrifuged at ×13,000*g* for 30 min, and the supernatant fractions were used for Western blot analysis as described previously [11]. Proteins (20 μg/lane) were separated using 10 % sodium dodecyl sulfate polyacrylamide gel electrophoresis and transferred to polyvinylidene fluoride membranes, and the membranes were blocked in PBS containing 3 % skim milk for 30 min. Each blot was incubated overnight at 4 °C with the primary antibody against goat anti-β-actin (Sigma-Aldrich) or goat anti-arginase I (Arg I) (marker of M2 cell, Santa Cruz Biotechnology, Inc.). After washing in PBS, membranes were incubated with horseradish peroxidase-conjugated secondary anti-goat immunoglobulin G (IgG) (GE Healthcare, Arlington Heights, IL, USA) for 2 h. Subsequent visualization was performed using an enhanced chemiluminescence system.

### Mixed glial culture and microglia enrichment

Primary mixed glia cultures were prepared from the spinal cords of 250-g adult SD rats. Briefly, the spinal cord was removed from the vertebral column, the meninges and blood vessels were carefully excised, and the spinal cord was finely chopped with scissors. The cell aggregations were further dissociated using 0.25 % trypsin/0.05 % EDTA and gentle trituration using a pipette, washed in DMEM containing 10 % fetal bovine serum, and centrifuged. The pellet was resuspended in culture medium, passed through a 70-μ nylon mesh, washed a second time, and centrifuged. After centrifugation, the cells were seeded at a density of 5 × 10^5^ cells/ml and incubated at 37 °C with 5 % CO_2_ for 48 h. After 48 h, non-adherent cells were removed, and fresh medium was added. For microglia enrichment, cultures were thoroughly shaken on an orbital shaker (120 rpm at room temperature). After 2 h, cells suspended in the culture medium were collected and centrifuged at 1500 rpm for 15 min at 4 °C. The cell pellet was resuspended and diluted with fresh culture medium to a final concentration of 5 × 10^4^ cells/ml, and the cell suspension was added to each well of a 48-well plate. After 20 min, non-adherent cells were discarded, and adherent cells were maintained in fresh culture medium. The enriched microglia were >85 % pure as determined by counting the OX42-positive cells and total cells stained with 4′,6′-diamidino-2-phenylindole (DAPI, Sigma-Aldrich).

### LPS stimulation and nitric oxide assay

Microglia were presensitized with 1 μg/ml lipopolysaccharide (LPS) in serum-free DMEM for 24 h. After LPS stimulation, the medium was replaced with DMEM or DMEM with 100 ng/ml DcR3.Fc for 24 h. Then, the culture medium was collected for the nitric oxide (NO) assay, and the cells were fixed with 4 % paraformaldehyde for immunofluorescence analysis. NO production was determined using the Griess reaction. Briefly, the culture medium was mixed with Griess reagents and incubated at room temperature for 10 min. The absorbance of the resultant products was measured at 540 nm. Sodium nitrite was used as a standard to calculate nitrogen dioxide concentrations.

### Cytokine measurement by reverse transcription real-time PCR

The primers for quantitative real-time polymerase chain reaction (qPCR) analysis are as follows: IL-4 (F-CGTCACTGACTGTAGAGAGC, R-GGGCTGTCGTTACATCCG), IL-10 (F-GTTGCCAAGCCTTGTCAGAA, R-TTTCTGGGCCATGGTTCTCT), and IL-13 (F-CTTGCCTTGGTGGTCTTG, R-TCTTCTGGTCTTGTGTGATG). Total RNA was extracted using TRIzol reagent (Invitrogen™) according to the manufacturer’s instructions. First-strand cDNA was synthesized using a RevertAid First-Strand cDNA Synthesis Kit (Thermo Scientific) as per the manufacturer’s instructions. The PCR reaction was performed in a LightCycler® System SW 3.5.3 (Roche Applied Science) under the following conditions: PCR mixtures were denatured at 95 °C for 5 min, followed by 45 cycles of 15 s at 95 °C, 30 s at 60 °C, and 30 s at 72 °C for amplification. The mRNA expression level of each target gene was normalized to the respective 18S rRNA expression.

### Immunohistochemistry

Fixed cells were permeabilized with 1 % Triton X-100, blocked with 1 % bovine serum albumin, and incubated first with primary antibodies overnight at 4 °C and then with secondary antibodies for 1 h at 37 °C. The primary antibody used was mouse anti-inducible nitric oxide synthase (iNOS) (1:500, BD). The secondary antibody used for fluorescence microscopy was Alexa 594-conjugated donkey anti-mouse IgG (Molecular Probes, Carlsbad, CA, USA), and nuclear staining was achieved using 1 μg/ml DAPI for 1 min. For in vivo analysis, rats were perfused 7 days post-injury as described above. Samples of spinal cords including the lesion site (2 cm in length) were removed from the experimental animals. The spinal cord was longitudinally sectioned (20 μm thick) and placed on slides for inflammatory marker analysis. The primary antibodies used were goat anti-Arg I (Santa Cruz Biotechnology, Inc.), goat anti-CD206 (R&D Systems), goat anti-IL-1β (R&D Systems), mouse anti-OX42 (marker of macrophages, BD Serotec, Oxford, UK), and mouse anti-RECA-1 (marker of blood vessels, BD Serotec, Oxford, UK). The secondary antibodies used for fluorescence microscopy were Alexa 594-conjugated donkey anti-goat IgG (Molecular Probes, Carlsbad, CA, USA), Alexa 488-conjugated donkey anti-mouse IgG (Molecular Probes, Carlsbad, CA, USA), and Alexa 594-conjugated donkey anti-mouse IgG (Molecular Probes, Carlsbad, CA, USA). Photographs were taken of the slides containing stained sections with a Zeiss LSM 7MP confocal microscope (Carl Zeiss, Oberkochen, Germany), and images were collected and arranged in Adobe Photoshop (Adobe Systems, Inc., San Jose, CA, USA). The antigens of Arg I, CD206, IL-1β, OX42, and RECA-1 were quantified using the NIH ImageJ 1.44d software in four longitudinal sections; three to four images were obtained through random sampling inside the epicenter area of each rat. The threshold values of antigen were maintained at constant levels in all analyzed images.

### Statistical analysis

The statistical analyses were performed with two-tailed Student’s *t* tests using GraphPad Prism. Differences with a *p* value <0.05 were considered significant.

## Results

### Improvement of hind limb functional recovery by DcR3.Fc

**Fig. 1:**
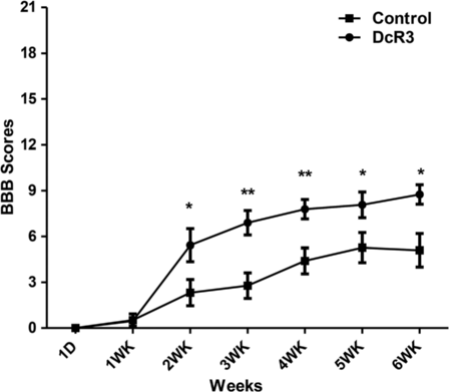
DcR3 treatment improved the hind limb functional recovery in SCI rats. Rats were contused at the T9 level of the spinal cord using the NYU impactor (10-g rod dropped from a height of 50 mm), followed by injection of 15 μg of DcR3.Fc in 5 μl of normal saline or 5 μl of normal saline only (control) at the injury epicenter. The functional recovery of the spinal cords was evaluated weekly using the BBB test. DcR3.Fc treatment yielded higher scores from the second week until the animals were euthanized (sixth week). (*p < 0.05, **p < 0.01, DcR3.Fc, *N* = 7; control, *N* = 10). The results are presented as means ± SEM

Because DcR3.Fc possesses anti-inflammatory activities and skews macrophage differentiation into M2 phenotypes, we asked whether administration of DcR3.Fc is beneficial to animals after spinal cord injury. To address this question, SD rats were subjected contusion injury using the NYU impactor at the level of the ninth thoracic vertebra, followed by the administration of DcR3.Fc into the injury epicenter. The contusive SCI resulted in immediate complete paralysis. Functional recovery was evaluated for up to 6 weeks using the BBB open-field score. A previous study showed that the BBB score was 5.1 ± 0.7 at the sixth week after injury in the same animal model, SD rats [33], and our study yielded a similar score for the control group (5.1 ± 1.097). Interestingly, DcR3.Fc-treated SCI rats showed higher BBB scores with better functional recovery of hind limb activities from the second week (5.4 compared with 2.3) continuing to the sixth week (8.8 compared with 5.1) after contusion injury (Fig. 1). This result suggests that DcR3.Fc is able to improve functional recovery after SCI.

### DcR3.Fc treatment reduces cavity formation and increases myelin sparing at lesion site

**Fig. 2:**
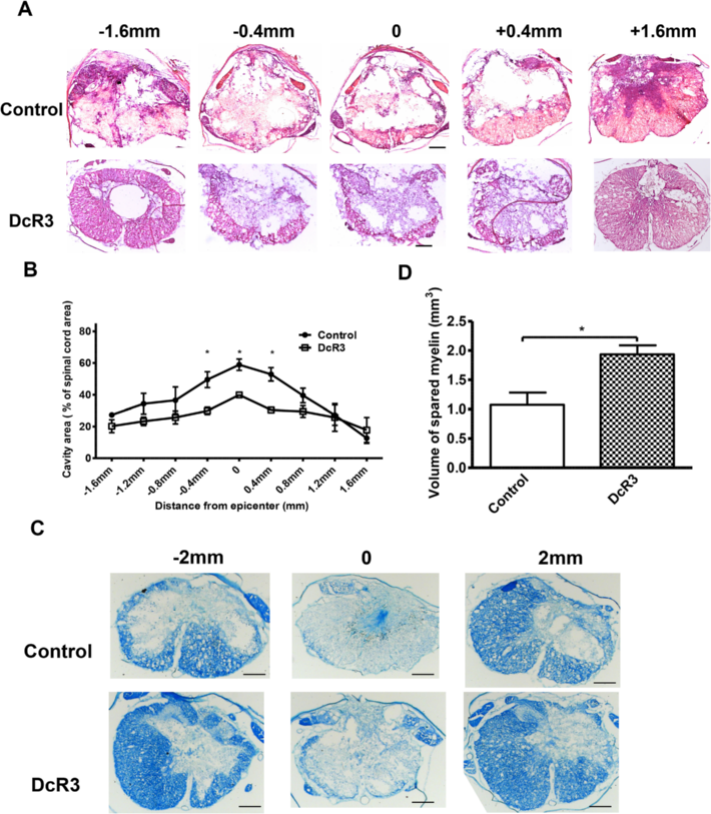
DcR3.Fc treatment reduces cavity formation and increases myelin sparing at the lesion site. Histological evaluation after DcR3.Fc treatment at the sixth week after SCI. **a** One of the representative results shows that DcR3.Fc-treated and control SCI rats were examined under microscope after H&E staining (scale bar: 250 μm). **b** Quantified empty space of the total spinal area from two groups to determine the percentage of the cavity area at the epicenter and the rostral to caudal (−1.6 to 1.6 mm, 0 mm indicates the epicenter) after injury. The cavity areas show that DcR3 treatment produced a smaller cavity area at the epicenter and the rostral to caudal (−0.4 to 0.4 mm) of the injury (*p < 0.05, *N* = 4 in each group). **c** LFB staining of the transverse spinal cord sections (−2, 0, and 2 mm) in DcR3.Fc-treated and control SCI rats (scale bar: 250 μm). **d** Quantified volumes of myelin sparing from −2 mm rostral to 2 mm caudal of the epicenter at the lesion site. DcR3.Fc-treated rats have higher tissue volume than control group. (*p < 0.05, DcR3.Fc, *N* = 4 in each group). The results are presented as means ± SEM

Contusion injury of the spinal cord not only causes immediate cell death but also triggers inflammatory reaction to cause additional cell loss, demyelination, and cavity formation in the later stage [31]. Thus, we asked whether DcR3.Fc administration could reduce cavity formation and increase myelin sparing. To address this question, the spinal cord was transversely sectioned from the rostral to the caudal ends, including the injury epicenters of the SCI rats at the sixth week after contusion injury. H&E staining was used to examine cavity size, while LFB staining was used to identify myelin sparing. The localization of injury epicenter was selected using the following criteria as described previously [34]: (1) the largest area of cavity relative to other tissue sections of the same animal and (2) the smallest area of myelin sparing. The sizes of wound cavities close to the lesion centers were much smaller in DcR3.Fc-treated rats after SCI. The differences in injured spinal cords were statistically significant at the center of the epicenter (39 vs. 59 %) as well as rostral (−0.4 mm; 29 vs. 49 %) to caudal (0.4 mm; 30 vs. 58 %) of the epicenter between the control and DcR3.Fc-treated groups (Fig. 2a, b). Furthermore, the total volumes of white matter sparing were measured in the LFB-stained transverse spinal cord sections (4 mm distance for the rostral-caudal spread including epicenter). As shown in Fig. 2c, d, higher volume of white matter sparing with more intact structure was observed in DcR3.Fc-treated rats than that of control group (1.934 vs. 1.075 mm^3^). These results suggested that DcR3.Fc treatment can significantly reduce tissue damage at the lesion site after SCI.

### Induction of anti-inflammatory cytokines by DcR3.Fc after SCI

**Fig. 3:**
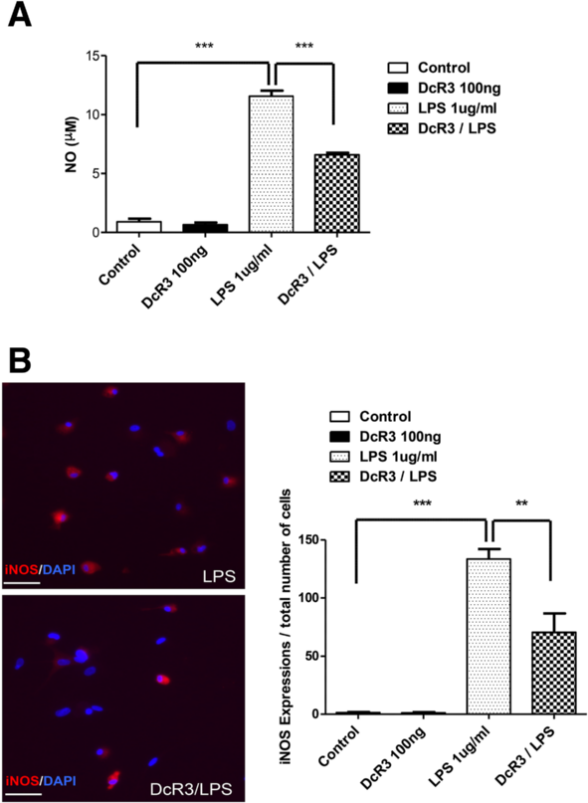
DcR3 treatment reduced LPS-induced NO production and iNOS expression in the primary microglia culture. **a** The production of NO was determined by measuring the nitrite amount produced by microglial cells after LPS stimulation. The NO production was elevated in microglial cells after LPS stimulation. In contrast, DcR3.Fc-treated microglia had significantly reduced NO production after LPS stimulation (***p < 0.001, *N* = 3). **b** Immunofluorescence staining of iNOS- and DAPI-positive cells in LPS-stimulated microglia with (*lower left*) or without (*upper left*) DcR3.Fc treatment. The average of the integrated optical density (IOD) of iNOS relative to the total cells (DAPI+ cells) in three or four random fields in each well was shown reduced in the DcR3.Fc-treated group (*right*, **p < 0.01, ***p < 0.001, *N* = 3). The results are presented as means ± SEM

**Fig. 4:**
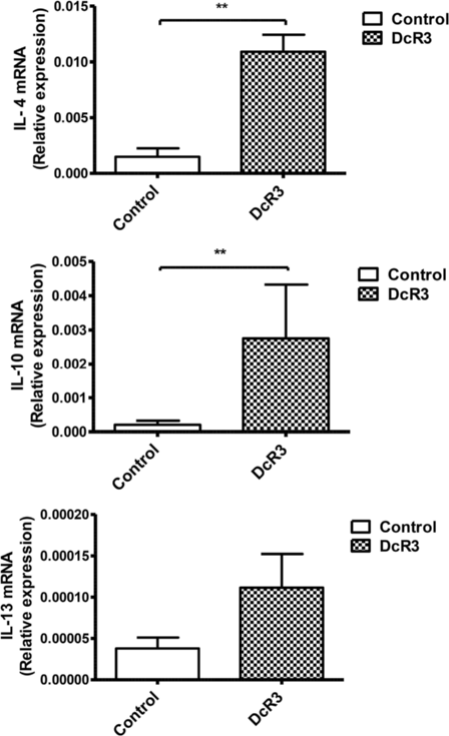
DcR3 treatment increased the expression of anti-inflammatory cytokines. Five millimeters of the spinal cord tissue from the lesion sites of DcR3.Fc-treated and control SCI rats was extracted on day 7 post-injury. Anti-inflammatory associated cytokines, namely IL-4, IL-10, and IL-13, were analyzed by qPCR. The target gene quantity was normalized to a reference gene (GAPDH) using the following formula: 2^−(Ct(target) − Ct(reference))^. The expression of anti-inflammatory cytokines, including IL-4 and IL-10, was significantly elevated at the lesion sites of DcR3-treated SCI rats (**p < 0.01, DcR3.Fc, *N* = 5; control, *N* = 6). The results are presented as means ± SEM

**Fig. 5:**
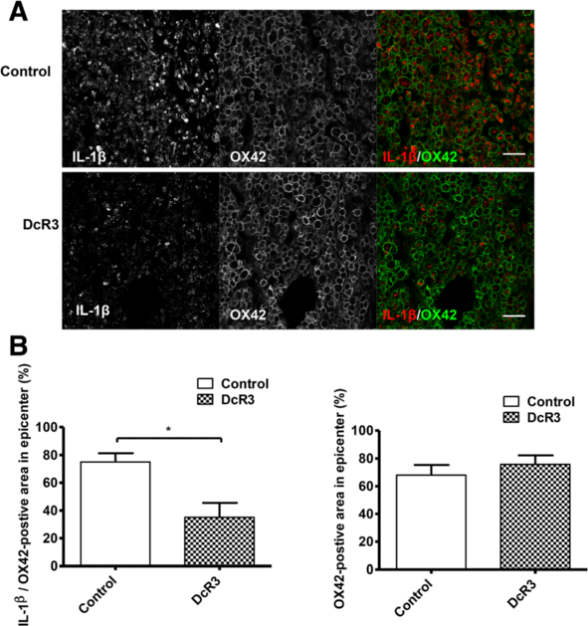
DcR3 treatment attenuates M1 macrophages in the lesion site of injured rat spinal cords. Expression of IL-1β at the lesion sites of DcR3.Fc-treated and control SCI rats. **a** Detection of IL-1β/OX42-positive cells in two groups by immunofluorescence staining (IL-1β, *red*; OX42, *green*; scale bar: 100 μm). **b** Quantification as a percentage of the IL-1β/OX42-positive area from the two groups in the lesion sites. The results showed that DcR3Fc-treated group had a lower percentage of IL-1β/OX42-positive area in the lesion site (*left*, *p < 0.05, *N* = 3). The results are presented as means ± SEM

SCI induces neuroinflammation and devastating neuronal death. DcR3 was shown to decrease NO production in monocyte-derived macrophages [25]; however, its modulatory effects on microglia have not been tested yet. To address this question, a microglia-enriched culture system (>85 % purity, Fig. 3a) was incubated with or without DcR3.Fc to test whether DcR3.Fc was able to reduce the NO production in microglia after LPS stimulation. We found that DcR3.Fc significantly reduced the NO production and iNOS expression in LPS-stimulated microglia (Fig. 3b). Because DcR3 is able to polarize inflammatory reactions toward the Th2-dominant response [35], we examined the expression of cytokines that are characteristic of the Th1 and Th2 responses. We found that the expression of the IL-4 and IL-10 was upregulated significantly at the lesion sites of DcR3.Fc-treated SCI rats on day 7 post-injury (Fig. 4). In contrast, the expression of the pro-inflammatory cytokine IL-1β (a marker of M1 macrophage) [36, 37] was downregulated at the lesion site of DcR3.Fc-treated SCI rat (Fig. 5a). Compared to the control group, DcR3.Fc-treated rats had a significantly lower percentage of IL-1β/OX42-positve area in the lesion sites of DcR3.Fc-treated rats at day 7 after contusion injury (Fig. 5b, left), even though the OX42-positve area were similar between the DcR3.Fc-treated and control groups (Fig. 5b, right). These results demonstrate that DcR3.Fc is able to upregulate anti-inflammatory cytokines and downregulate IL-1β expression in SCI animals.

### DcR3 recruited M2 macrophages to the lesion site of injured spinal cord

**Fig. 6:**
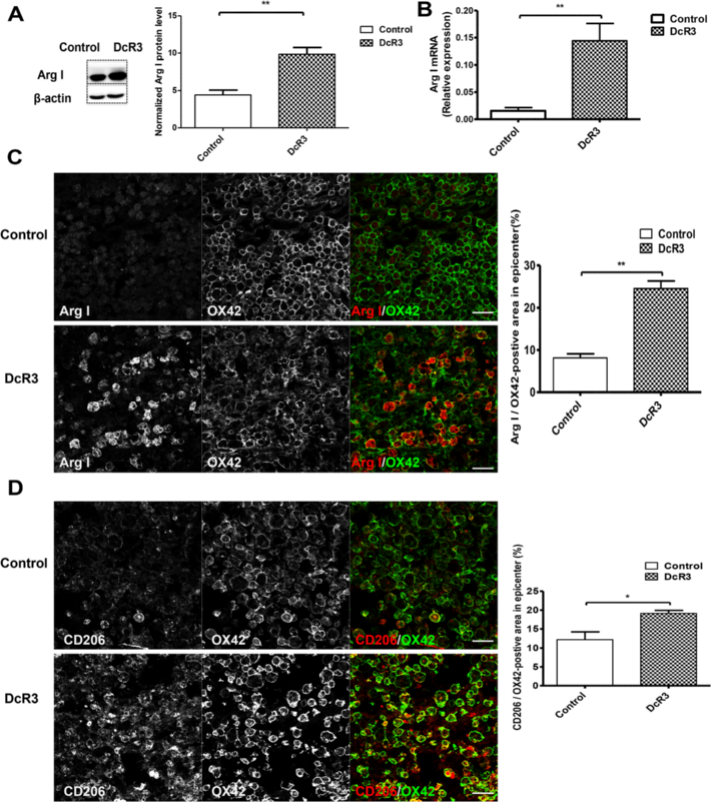
Recruitment of M2 macrophages at the lesion site of injured rat spinal cords after DcR3.Fc treatment. Expressions of Arg I and CD206 at the lesion sites of DcR3.Fc-treated and control SCI rats. **a**, **b** Detection of the Arg I expressions at the lesion sites of the two groups by Western blot analysis (**a** **p < 0.01, *N* = 3) and qPCR (**b** **p < 0.01, *N* = 5). **c**, **d** Detection of Arg I/OX42-positive cells **(c)** and CD206/OX42-positive cells **(d)** at the lesion sites of the two groups of rats by immunofluorescence staining (*left*, scale bar: 100 μm), and quantification as the percentage of Arg I/OX42-positive area (**c** *right*, **p < 0.01, *N* = 3) and CD206/OX42-positive area in the lesion area (**d** *right*, *p < 0.05, *N* = 3). The results are presented as means ± SEM

M2 macrophages perform immune-resolving functions and participate in tissue repair. Because DcR3 was shown to promote M2 macrophage differentiation, we further asked whether DcR3 recruited M2 macrophages to the lesion sites of SCI rats. To address this question, we evaluated the expression of the M2 marker Arg I, which is involved arginine and polyamines production and is beneficial to neuronal survival and regeneration in the CNS [38]. As shown in Fig. 6, the upregulated Arg I expression was observed at the lesion sites of DcR3-treated SCI rats on day 7 after contusion injury, as determined by Western blot and qPCR analyses (Fig. 6a, b). Immunofluorescence staining of Arg I was increased and co-localized with OX42 (microglia/macrophage marker) in DcR3-treated rats (Fig. 6c, left). In addition, CD206 (M2 marker) was also co-localized with OX42-positive cells (Fig. 6d, left). Compared with the control, DcR3.Fc-treated rats had significantly higher ratios of Arg I/OX42 and CD206/OX42-positve areas in the lesion area on day 7 after contusion injury (Fig. 6c, d, right). These observations suggest that DcR3.Fc skewed microglia into the M2 phenotype at the lesion site after contusion injury.

### DcR3 treatment promotes angiogenesis at the lesion site

**Fig. 7:**
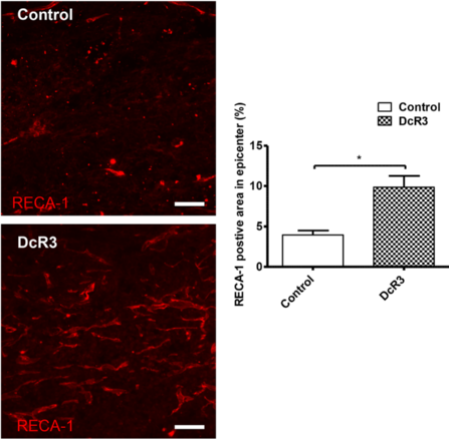
DcR3 treatment promoted angiogenesis at the lesion site. Population of blood vessels (RECA-1-positive, *red*) at the lesion sites of DcR3.Fc-treated and control SCI rats (*left*, scale bar: 100 μm). Quantification as the percentage of the RECA-1-positive area related to the total area in three random fields at the lesion site of each section (*right*, *p < 0.05, *N* = 3). The results are presented as means ± SEM

Angiogenesis occurs following SCI, and the extent of angiogenesis correlates with tissue repair and neuronal regeneration. Therefore, we investigated the DcR3-induced angiogenesis at the lesion site by examining the expression of RECA-1, a specific marker for rat endothelial cells of blood vessels. Compared with the control group, higher expression of RECA-1 (longitudinal spinal cord section) in the epicenter at 7 days after SCI was noted in DcR3.Fc-treated rats (Fig. 7), suggesting enhanced angiogenesis in the lesion site after DcR3.Fc treatment. All the observations above suggest that DcR3.Fc is able to skew microglia into the M2 phenotype and promote angiogenesis at the SCI lesion site, resulting in the preservation of a greater amount of spinal cord tissue and the enhancement of functional recovery after SCI.

## Discussion

The aim of this research was to study the beneficial effects of DcR3 in the injured spinal cord, specifically with regard to the effect of the immunomodulatory function on DcR3.

The M1 macrophages within the epicenter of an injured spinal cord orchestrate inflammatory responses, leading to further devastating neuronal cell death and the loss of neurons, oligodendrocytes, and myelin. In contrast, M2 macrophages promote angiogenesis, tissue repair, and axonal growth during acute inflammation [14]. Therefore, regulation of macrophage differentiation into an M1 or M2 phenotype in the microenvironment would determine whether infiltrating macrophages aggravate secondary injury or promote wound repair [8, 10, 11]. We have shown that DcR3.Fc is able to downregulate the expression of pro-inflammatory cytokines (IL-1β, tumor necrosis factor alpha, and IL-6) and iNOS in macrophages [24]. In addition, we also showed that DcR3 is a potent immunomodulator that skews macrophage differentiation into the M2 phenotype in DcR3-trangenic mice [25, 26]. In this study, we further demonstrate that injection of DcR3.Fc at the lesion site can skew microglia/macrophages into the M2 phenotype and promote tissue repair and functional recovery after SCI in vivo. This observation further confirms the potent immunomodulatory effect of DcR3 and strengthens the argument that DcR3.Fc-mediated M2 macrophage differentiation is beneficial in SCI.

The other immunomodulatory effect of DcR3 occurs via the promotion of Th2-dominant responses. We have shown that DcR3-treated dendritic cells are able to skew host immunity to Th2-dominant responses [22, 35], and this observation is consistent with the upregulation of IL-4 and IL-10 in the lesion sites of DcR3-treated SCI rats (Fig. 4). IL-10 not only inhibits Th1 response but also downregulates the expression of IL-1β, NO synthase, and reactive oxygen species. In addition, IL-4 is able to promote M2 macrophage differentiation and further promote IL-10 secretion [39]. In our previous and this study, we demonstrated that DcR3.Fc suppressed the NO and iNOS expression in primary cultures of microglia (Fig. 3) and monocyte-derived macrophages in vitro [24], and it significantly increases the IL-4 and IL-10 expression at lesion sites on day 7 after SCI rats (Fig. 4). Furthermore, the IL-1β expression in microglia/macrophage at the lesion site was downregulated by DcR3.Fc treatment (Fig. 5), whereas IL-13 expression was not affected under the same condition. This observation is in consistent with our previous observation that IL-4 and IL-10 but not IL-13 were upregulated after antigen stimulation in splenocytes isolated from DcR3 transgenic mice [35]. Besides, a previous study showed that IL-4 mRNA expression was upregulated while the IL-13 mRNA was downregulated at the lesion site in the first 3 days after SCI [40], suggesting that IL-13 may not be involved in the spontaneous repair after SCI. Thus, DcR3-mediated tissue/neuronal protection occurs via the upregulation of IL-4 and IL-10 but not of IL-13 at the lesion site in SCI.

After SCI, acute necrosis and secondary damage resulted in cavitation and demyelination, which persist progressively over time and increase the extent of damage after injury [41]. It has been reported that the functional recovery of thoracic SCI correlated inversely with reduced cavity size [41] and increase of white matter sparing [42], and thoracic contusion injury with few white matter sparing is sufficient to help locomotor movements. This observation suggests that increase of spared tissue at lesion site may contribute to the improvement of hind limb locomotor function after SCI [29]. Because DcR3.Fc treatment reduced the extent of cavitation and increased white matter sparing at lesion area, the improvement of functional recovery after severely thoracic SCI seems to be attributed to DcR3-mediated tissue repairing (Figs. 1 and 2).

DcR3 is able to enhance angiogenesis via neutralizing the angiogenesis inhibitor (TL1A) [19], in addition to promoting the Th2-predominant reaction and M2 differentiation. Furthermore, M2 macrophages are able to promote angiogenesis [43], which reduces the secondary damage after SCI by increasing the delivery of oxygen and nutrients to sites of regeneration [44]. In this study, we demonstrated that the intrathecal administration of DcR3.Fc upregulated the expression of RECA-1 (Fig. 7), which correlated with the increase in tissue preservation at the lesion area. Thus, DcR3 may increase angiogenesis via neutralizing endogenous TL1A and promoting M2 macrophage differentiation.

These observations suggest that DcR3.Fc is a potent pleiotropic immunomodulator, and the administration of DcR3.Fc in lesion site is a promising approach to reduce tissue damage and enhances the functional recovery of locomotion after SCI.

## Conclusions

Several strategies, including cell therapy, molecular therapy, and combinatorial treatment, have been developed in SCI animal models [21, 45, 46]. However, only a few approaches can be applied in clinical trials. In the present study, we demonstrated that DcR3.Fc can modulate the macrophage response to SCI to facilitate tissue sparing and functional recovery. Thus, DcR3.Fc may become a promising therapeutic agent for SCI patients in the future. However, more studies are needed to confirm the effectiveness as well as to determine the most optimal therapeutic window, dosages, and timing for the administration of DcR3.Fc after SCI. Moreover, increasing evidence demonstrates that combinatorial strategies employing multiple agents are able to further improve locomotor functions after SCI [47]. Multiple injections of DcR3.Fc in conjunction with pertinent neurotrophic factors, scar-resolving agents, or other axonal growth-promoting agents may facilitate the development of a better strategy for SCI treatment in the future.

## Abbreviations

Arg I, arginase I; BBB, Basso, Beattie, and Bresnahan; CNS, central nervous system; DAPI, 4′,6′-diamidino-2-phenylindole; DcR3, decoy receptor 3; DcR3.Fc, recombinant protein comprising DcR3; H&E, hematoxylin and eosin; IgG, immunoglobulin G; IL-, interleukin-; iNOS, inducible nitric oxide synthase; LFB, Luxol fast blue; LPS, lipopolysaccharide; New York University, NYU; NO, nitric oxide; PBS, phosphate-buffered saline; qPCR, quantitative real-time polymerase chain reaction; SCI, spinal cord injury; SD, Sprague-Dawley; TNFRSF, tumor necrosis factor receptor superfamily member
